# Development and Validation of a Novel Nomogram to Predict Improved Left Ventricular Ejection Fraction in Patients With Heart Failure After Successful Percutaneous Coronary Intervention for Chronic Total Occlusion

**DOI:** 10.3389/fcvm.2022.864366

**Published:** 2022-04-14

**Authors:** Lulu Yang, Huan Li, Guangli Guo, Jiaqi Du, Zhengyang Hao, Lingyao Kong, Huiting Shi, Xiaofang Wang, Yanzhou Zhang

**Affiliations:** Department of Cardiology, The First Affiliated Hospital of Zhengzhou University, Zhengzhou, China

**Keywords:** chronic total occlusion, heart failure, left ventricular ejection fraction, nomogram, prediction model

## Abstract

**Background:**

Heart failure with improved left ventricular ejection fraction (HFiEF) is linked to a good clinical outcome. The purpose of this study was to create an easy-to-use model to predict the occurrence of HFiEF in patients with heart failure (HF), 1 year after successful percutaneous coronary intervention (PCI) for chronic total occlusion (CTO) (CTO PCI).

**Methods:**

Patients diagnosed with HF who successfully underwent CTO PCI between January 2016 and August 2019 were included. To mitigate the effect of residual stenosis on left ventricular (LV) function, we excluded patients with severe residual stenosis, as quantitatively measured by a residual synergy between PCI with Taxus and Cardiac Surgery score (rSS) of >8. We gathered demographic data, medical history, angiographic and procedural characteristics, echocardiographic parameters, laboratory results, and medication information. The least absolute shrinkage and selection operator (LASSO) and multivariate logistic regression models were used to identify predictors of HFiEF 1 year after CTO revascularization. A nomogram was established and validated according to the area under the receiver operating characteristic curve (AUC) and calibration curves. Internal validation was performed using bootstrap resampling.

**Results:**

A total of 465 patients were finally included in this study, and 165 (35.5%) patients experienced HFiEF 1 year after successful CTO PCI. According to the LASSO regression and multivariate logistic regression analyses, four variables were selected for the final prediction model: age [odds ratio (OR): 0.969; 95% confidence interval (CI): 0.952–0.988; *p* = 0.001], previous myocardial infarction (OR: 0.533; 95% CI: 0.357–0.796; *p* = 0.002), left ventricular end-diastolic dimension (OR: 0.940; 95% CI: 0.910–0.972; *p* < 0.001), and sodium glucose cotransporter two inhibitors (OR: 5.634; 95% CI: 1.756–18.080; *p* = 0.004). A nomogram was constructed to present the results. The C-index of the model was 0.666 (95% CI, 0.613–0.719) and 0.656 after validation. The calibration curve demonstrated that the nomogram agreed with the actual observations.

**Conclusions:**

We developed an simple and effective nomogram for predicting the occurrence of HFiEF in patients with HF, 1 year after successful CTO PCI without severe residual stenosis.

## Introduction

Left ventricular ejection fraction (LVEF) is a reliable measure of cardiac function and is commonly used as a prognostic parameter for risk stratification by clinicians. Heart failure with improved LVEF (HFiEF) represents an improvement in left ventricular (LV) function and is closely associated with favorable clinical outcomes (1). Therefore, to improve long-term prognosis by therapeutic optimization, it is crucial to identify the predictors of HFiEF.

Chronic total occlusion (CTO) predisposes patients with heart failure (HF) to adverse clinical events (2). Revascularization of CTO is reportedly beneficial for LV function, symptomatic relief, and survival outcomes in patients with HF (3–5). In real-world clinical practice in China, many patients tend to prefer percutaneous coronary intervention (PCI) for CTO revascularization rather than coronary artery bypass grafting (CABG), because of the higher surgical risk associated with the latter. However, residual stenosis is quite common and even unavoidable in PCI, despite remarkable advancements in technology as well as the available techniques (6, 7). In this regard, several studies have assessed the relationship between the extent of revascularization, quantified by the residual Synergy between PCI with Taxus and Cardiac Surgery (SYNTAX) score (rSS), and the occurrence of adverse clinical outcomes, and determined that an rSS of ≤ 8 was reasonable for PCI (8, 9). However, opening CTO and achieving a low rSS require not only abundant manpower, resources, and time, but are also associated with exposure to high doses of radiation for both patients and cardiologists during PCI, particularly in multivessel disease. It is still unclear which individuals are suitable candidates for this treatment strategy, aimed at improving the prognosis while avoiding high surgical risk. Moreover, existing nomograms either focus on patients with acute heart failure (AHF) or assess survival (10–14). Therefore, we aimed to build a prognostic nomogram for the prediction of HFiEF, 1 year after successful CTO PCI with an rSS ≤ 8 in CTO patients with HF to optimize the treatment options.

## Methods

### Patient Selection

This was a retrospective study of patients with HF who underwent successful CTO PCI at the First Affiliated Hospital of Zhengzhou University, Henan, China, between January 2016 and August 2019. The inclusion criteria were as follows: (1) at least one CTO lesion with a diameter of >2 mm located in the proximal or middle segment of the epicardial artery; (2) LVEF <50%, as confirmed by echocardiography, single-photon emission computed tomography (SPECT), or cardiovascular magnetic resonance (CMR); and (3) symptomatic angina and/or a functional ischemia test result. The exclusion criteria were as follows: (1) only medical therapy was administered to the patients, (2) CTO located in a branch vessel or a distal lesion, (3) CABG history, (4) an ST-segment elevation acute myocardial infarction (MI) within 48 h, and (6) the presence of a malignant tumor. The Scientific Research and Clinical Trial Ethics Committee of the First Affiliated Hospital of Zhengzhou University approved this study and waived the need to obtain informed consent from the patients, considering the retrospective nature of the study.

### Data Collection

The predictors included in our study were as follows: demographic data (e.g., age and sex), comorbidities (e.g., hypertension, diabetes mellitus, atrial fibrillation, and renal insufficiency), laboratory results (e.g., fasting blood glucose, low-density lipoprotein, blood urea nitrogen, and serum creatinine), echocardiographic parameters (e.g., left ventricular ejection fraction, left ventricular end-diastolic dimension, and stroke volume), angiographic and procedural details (e.g., three-vessel disease, left main disease, and multiple CTOs), and medications used at discharge (e.g., statins, diuretics, aldosterone antagonists, and digoxin) at baseline. We performed the first echocardiography on admission. Similarly, we recorded the first laboratory test results within 48 h of admission. The primary endpoint was the occurrence of HFiEF 1 year after successful CTO PCI.

### Definitions

CTO was defined as a complete luminal obstruction with no antegrade flow or with antegrade or retrograde filling by collateral circulation for an estimated duration of at least 3 months, based on previous clinical data. HF was defined as an LVEF of <50% with dyspnea or equivalent symptoms. CTO PCI success was defined as the opening of CTO lesions and stent placement without immediate angiographic complications in the first or any subsequent attempts. For patients with multiple CTO lesions, CTO PCI success was defined as the opening of all CTO lesions. HFiEF was defined as an absolute LVEF improvement of ≥10% at 1 year. For those with a baseline LVEF of <40%, the LVEF had to improve by up to 40%.

### Statistical Analysis

All statistical analyses were carried out using R software and SPSS 22.0 (IBM Corp., Armonk, NY, USA). The mean ± standard deviation (SD) or median (interquartile range; IQR) has been used to express continuous variables. Categorical data are reported as counts or percentages. For data dimensionality reduction and variable selection, least absolute shrinkage and selection operator (LASSO) regression analysis was used. The lambda values were chosen after a 10-fold cross validation. Subsequently, multivariate logistic regression was used to identify independent factors influencing HFiEF. Then a nomogram was constructed. The concordance index (C-index) was calculated to assess the discriminative ability of the model *via* bootstrap resampling 1,000 times, and a receiver operating characteristic curve (ROC) was constructed. Calibration of the model was assessed according to a calibration curve, with 1,000 resamplings. A decision curve analysis (DCA) was performed to estimate the clinical benefit of the model. A two-tailed *p* < 0.05 was considered to denote statistical significance.

## Results

### Population Characteristics

**Figure 1:**
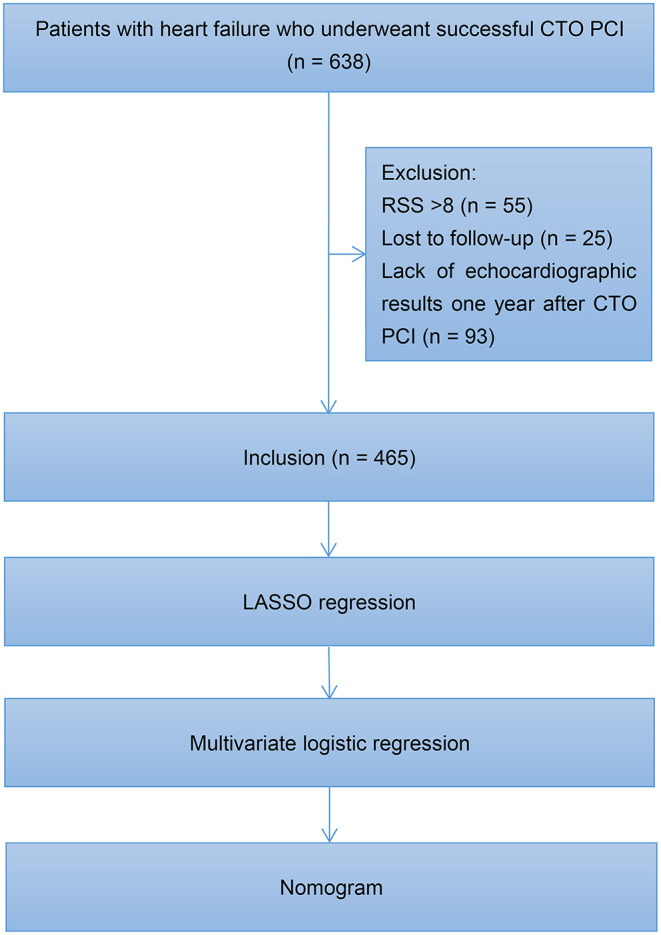
Study flowchart. CTO, chronic total occlusion; PCI, percutaneous coronary intervention; RSS, residual Synergy Between Percutaneous Coronary Intervention with Taxus and Cardiac Surgery score; LASSO, least absolute shrinkage and selection operator.

Figure 1 illustrates the study flowchart. A total of 638 patients diagnosed with HF underwent successful CTO PCI at our center between January 2016 and August 2019. One-year echocardiographic outcomes were available for 465 patients who were included in the final analysis. Among the 465 patients, 165 (35.5%) had HFiEF. These subjects were divided into two groups: HFiEF (*n* = 165) and non-HFiEF (*n* = 300) groups. The clinical characteristics of the two groups are summarized in Table 1. Compared with the non-HFiEF group, the HFiEF group had younger patients and a lower prevalence of previous myocardial infarction (MI), lower levels of N-terminal precursor B-type diuretic peptide (NT-proBNP), smaller left ventricular end-diastolic diameter (LVEDD), higher levels of estimated glomerular filtration rate (eGFR) and total cholesterol, as well as higher sodium glucose cotransporter 2 inhibitors (SGLT2i) use.

**Table 1:** Clinical characteristics.

	**Non-HFiEF** **(*n* = 300)**	**HFiEF** **(*n* = 165)**	***p-*value**
**Demographic data**			
Age, years	63 (54, 71)	60 (51, 67)	0.003^*^
Male, *n* (%)	233 (77.7)	127 (77.0)	0.863
**Past medical history**			
Diabetes mellitus, *n* (%)	94 (31.3)	45 (27.3)	0.360
Hypertension, *n* (%)	151 (50.3)	77 (46.7)	0.449
Atrial fibrillation, *n* (%)	7 (2.3)	4 (2.4)	1.000
PAD, *n* (%)	10 (3.3)	2 (1.2)	0.283
COPD, *n* (%)	9 (3.0)	2 (1.2)	0.371
Stroke, *n* (%)	52 (17.3)	22 (13.3)	0.259
Renal insufficiency, *n* (%)	33 (11.0)	15 (9.1)	0.517
Current smoking, *n* (%)	137 (45.7)	67 (40.6)	0.293
Previous myocardial infarction, *n* (%)	177 (59.0)	72 (43.6)	0.001^*^
Previous PCI	39 (13.0)	18 (10.9)	0.511
ACS, *n* (%)	96 (32.0)	56 (33.9)	0.670
NYHA≥ grade III, *n* (%)	128 (42.7)	69 (41.8)	0.859
**Angiographic and procedural characteristics**			
Baseline SYNTAX score	24 (19.30)	24 (20.29)	0.928
Residual SYNTAX score	1 (0.4)	0 (0.3)	0.225
Left arterial dominance, *n* (%)	87 (29.0)	52 (31.5)	0.571
Left main disease, *n* (%)	33 (11.0)	12 (7.3)	0.193
2-vessel disease, n (%)	104 (34.7)	54 (32.7)	0.673
3-vessel disease, *n* (%)	167 (55.7)	92 (55.8)	0.985
CTO location in LAD, *n* (%)	149 (49.7)	90 (54.5)	0.314
CTO location in LCX, *n* (%)	65 (21.7)	38 (23.0)	0.735
CTO location in RCA, *n* (%)	142 (47.3)	66 (40.0)	0.128
Multiple CTOs, *n* (%)	51 (17.0)	28 (17.0)	0.993
J-CTO score≥ 3, *n* (%)	40 (13.3)	13 (7.9)	0.077
Well-developed collateral flow, *n* (%)	276 (92.0)	157 (95.2)	0.199
**Echocardiographic parameters**			
LVEF, %	45 (39, 48)	45 (39, 48)	0.146
LVEDD, mm	56 (52, 60)	53 (49, 59)	0.001^*^
LVESV, ml	86 (68, 110)	85 (67, 107)	0.455
LVEDV, ml	154 (128, 184)	149 (125, 180)	0.257
SV, ml	67 (59, 76)	65 (56, 74)	0.062
**Laboratory results**			
TG, mmol/L	1.33 (1.03, 1.82)	1.35 (1.03, 1.87)	0.466
TC, mmol/L	3.47 (2.97, 4.16)	3.78 (3.12, 4.37)	0.044^*^
HDL-C, mmol/L	0.96 (0.83, 1.12)	0.97 (0.80, 1.16)	0.743
LDL-C, mmol/L	1.72 (1.59, 2.36)	1.75 (1.58, 2.58)	0.723
Fasting glucose, mmol/L	5.2 (4.5, 7.5)	5.3 (4.6, 7.4)	0.496
HbA1c, %	6.0 (5.6, 6.8)	6.0 (5.6, 6.8)	0.744
AST, U/L	25 (17, 40)	27 (19, 45)	0.130
ALT, U/L	24 (17, 43)	26 (18, 46)	0.223
Serum creatinine, umol/L	78 (67, 90)	73 (65, 88)	0.068
BUN, mmol/L	5.7 (4.6, 7.1)	5.4 (4.6, 6.6)	0.246
eGFR, ml/min/1.73 m^2^	88 (74, 97)	92 (79, 103)	0.004^*^
CRP, mg/L	3.2 (1.0, 8.5)	2.4 (0.9, 8.3)	0.700
NT-proBNP, pg/ml	1,494 (506, 2,935)	1,083 (420, 2,432)	0.042^*^
**Medications at discharge**			
Aspirin, *n* (%)	291 (97.0)	159 (96.4)	0.710
Clopidogrel, *n* (%)	111 (37.0)	49 (29.7)	0.113
Ticagrelor, *n* (%)	189 (63.0)	116 (70.3)	0.113
Statins, *n* (%)	300 (100)	165 (100)	-
ACEI/ARB, *n* (%)	280 (93.3)	156 (94.5)	0.605
Beta-blockers, *n* (%)	266 (88.7)	155 (93.9)	0.063
Aldosterone antagonists, *n* (%)	231 (77.0)	124 (75.2)	0.654
Diuretics, *n* (%)	180 (60.0)	92 (55.8)	0.374
Digoxin, *n* (%)	40 (13.3)	20 (12.1)	0.709
Nitrates, *n* (%)	67 (22.3)	34 (20.6)	0.666
SGLT2i, *n* (%)	4 (1.3)	13 (7.9)	<0.001^*^
ARNI, *n* (%)	4 (1.3)	3 (1.8)	0.703
Insulin, *n* (%)	29 (9.7)	12 (7.3)	0.384
Ivabradine, *n* (%)	4 (1.3)	1 (0.6)	0.660

*HFiEF, heart failure with improved ejection fraction; COPD, chronic obstructive pulmonary disease; PCI, percutaneous coronary intervention; ACS, acute coronary syndrome; NYHA, New York Heart Association; SYNTAX, Synergy Between Percutaneous Coronary Intervention with Taxus and Cardiac Surgery; PAD, peripheral artery disease; CTO, chronic total occlusion; LAD, left anterior descending artery; LCX, left circumflex artery; RCA, right coronary artery; J-CTO, Japanese multicenter registry; LVEF, left ventricular ejection fraction; LVEDD, left ventricular end-diastolic dimension; LVESV, left ventricular end-systolic volume; LVEDV, left ventricular end-diastolic volume; SV, stroke volume; TG, triglyceride; TC, total cholesterol; LDL-C, low-density lipoprotein cholesterol; HDL-C, high–density lipoprotein cholesterol; AST, aspartate aminotransferase; ALT, alanine transaminase; BUN, blood urea nitrogen; eGFR, estimated glomerular filtration rate; CRP, C-reaction protein; NT-proBNP, N-terminal precursor B-type diuretic peptide; ACEI, angiotensin converting enzyme inhibitor; ARB, angiotensin receptor blockers; SGLT2i, sodium glucose cotransporter 2 inhibitors; ARNI, angiotensin receptor neprilysin inhibition*.

* *p < 0.05*.

### LASSO Regression Analysis

**Figure 2:**
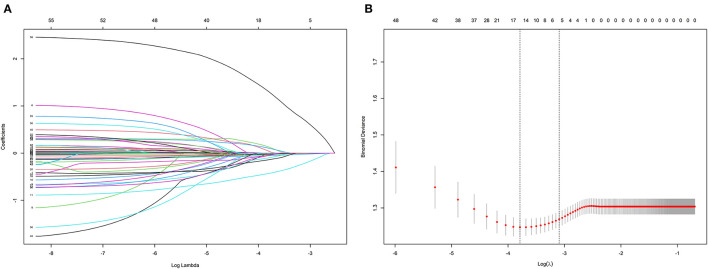
Clinical feature selection according to the least absolute shrinkage and selection operator (LASSO) regression analysis. **(A)** LASSO coefficient profiles of the 53 candidate predictors. **(B)** Turing parameter (λ) selection in the LASSO model using 10-fold cross-validation.

A total of 53 associated variables, including demographic data, past medical history, angiographic and procedural characteristics, echocardiographic parameters, laboratory results, and medications at discharge, were initially entered into the LASSO regression algorithm by 10-fold cross validation to identify the prognostic factors of HFiEF. Five potential variables with non-zero coefficients were selected: age (coefficient, −0.0080), previous MI (coefficient, −0.2080), eGFR (coefficient, 0.0007), LVEDD (coefficient, −0.0199), and SGLT2i (coefficient, 0.6890) (Figure 2A). Figure 2B depicts the changes in the LASSO coefficients.

### Multivariate Logistic Regression Analysis

The five predictors selected by LASSO were screened using multivariate logistic regression analysis to determine the independent factors predicting HFiEF at 1 year. Four variables were included in the final model. Table 2 shows the β-coefficients, standard error (SE), odds ratios (OR), 95% confidence intervals (CI), and *p*-values for each of the variables: age (OR: 0.969; 95% CI: 0.952–0.988; *p* = 0.001), previous MI (OR: 0.533; 95% CI: 0.357–0.796; *p* = 0.002), LVEDD (OR: 0.940; 95% CI: 0.910–0.972; *p* < 0.001), and SGLT2i (OR: 5.634; 95% CI: 1.756–18.080; *p* = 0.004).

**Table 2:** Multivariate analysis of HFiEF after 1 year.

**Variable**	**β**	**Standard error**	**Odds ratio (95% CI)**	***p-*value**
Age	−0.031	0.010	0.969 (0.952–0.988)	0.001^*^
Previous MI	−0.628	0.204	0.533 (0.357–0.796)	0.002^*^
LVEDD	−0.062	0.017	0.940 (0.910–0.972)	<0.001^*^
SGLT2i	1.729	0.595	5.634 (1.756–18.080)	0.004^*^

*HFiEF, heart failure with improved ejection fraction; CI, confidence interval; MI, myocardial infarction; LVEDD, left ventricular end-diastolic dimension; SGLT2i, sodium glucose cotransporter 2 inhibitors*.

* *p < 0.05*.

### Construction of a Prognostic Nomogram

**Figure 3:**
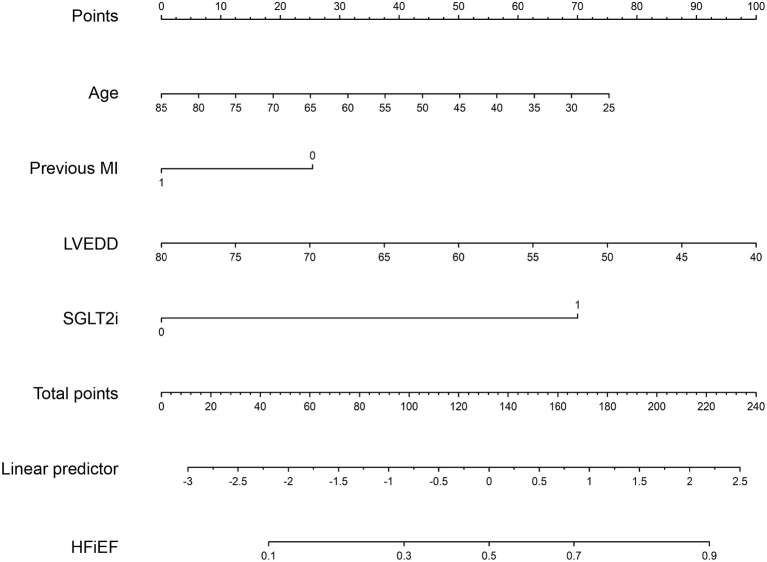
A nomogram to predict the probability of HFiEF 1 year after successful CTO PCI in patients with heart failure. MI, myocardial infarction; LVEDD, left ventricular end-diastolic dimension; SGLT2i, sodium glucose cotransporter 2 inhibitors.

A nomogram was constructed to predict HFiEF 1 year after successful CTO PCI in HF patients by incorporating the three predictive factors (age, previous MI, and LVEDD). The details of the scoring model are presented in Figure 3. The presence or level of each predictive factor was assigned one point, and the points for each individual factor were summed up to obtain the total.

### Evaluation and Validation of the Nomogram

**Figure 4:**
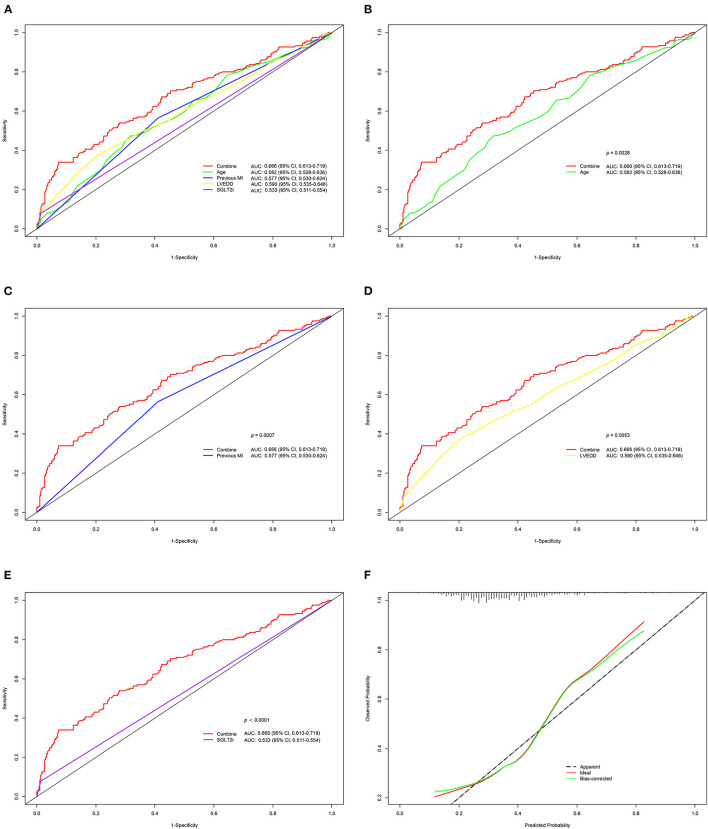
Validation of the nomogram for HFiEF at 1 year. **(A–E)** Comparison of ROC and the area under the curve (AUC) for the HFiEF nomogram with each index. **(F)** Calibration plot for the 1,000-time resampling bootstrap analysis.

We further assessed the discrimination and calibration of the model. The nomogram's C-index for predicting postoperative HFiEF was 0.666 (95% CI, 0.613–0.719) and was corrected to 0.656 by bootstrapping validation, which showed a moderate predictive accuracy. The ROC curves of the model and each index are presented in Figure 4A. The pooled area under the ROC of the nomogram was 0.666 (95% CI, 0.613–0.719), and the AUC was 0.582 (95% CI, 0.528–0.636) for age, 0.577 (95% CI, 0.530–0.624) for previous MI, 0.590 (95% CI, 0.535–0.646) for LVEDD, and 0.533 (95% CI, 0.511–0.554) for SGLT2i. Moreover, we compared the AUC between the nomogram and each predictive factor. The AUC of nomogram was significantly higher than that of age (*p* = 0.0028), previous MI (*p* = 0.0007), LVEDD (*p* = 0.0053), and SGLT2i (*p* < 0.0001) (Figures 4B–E). On the other hand, the calibration curve revealed that the predicted and actual probabilities for predicting HFiEF were in good agreement (Figure 4F).

### DCA

**Figure 5:**
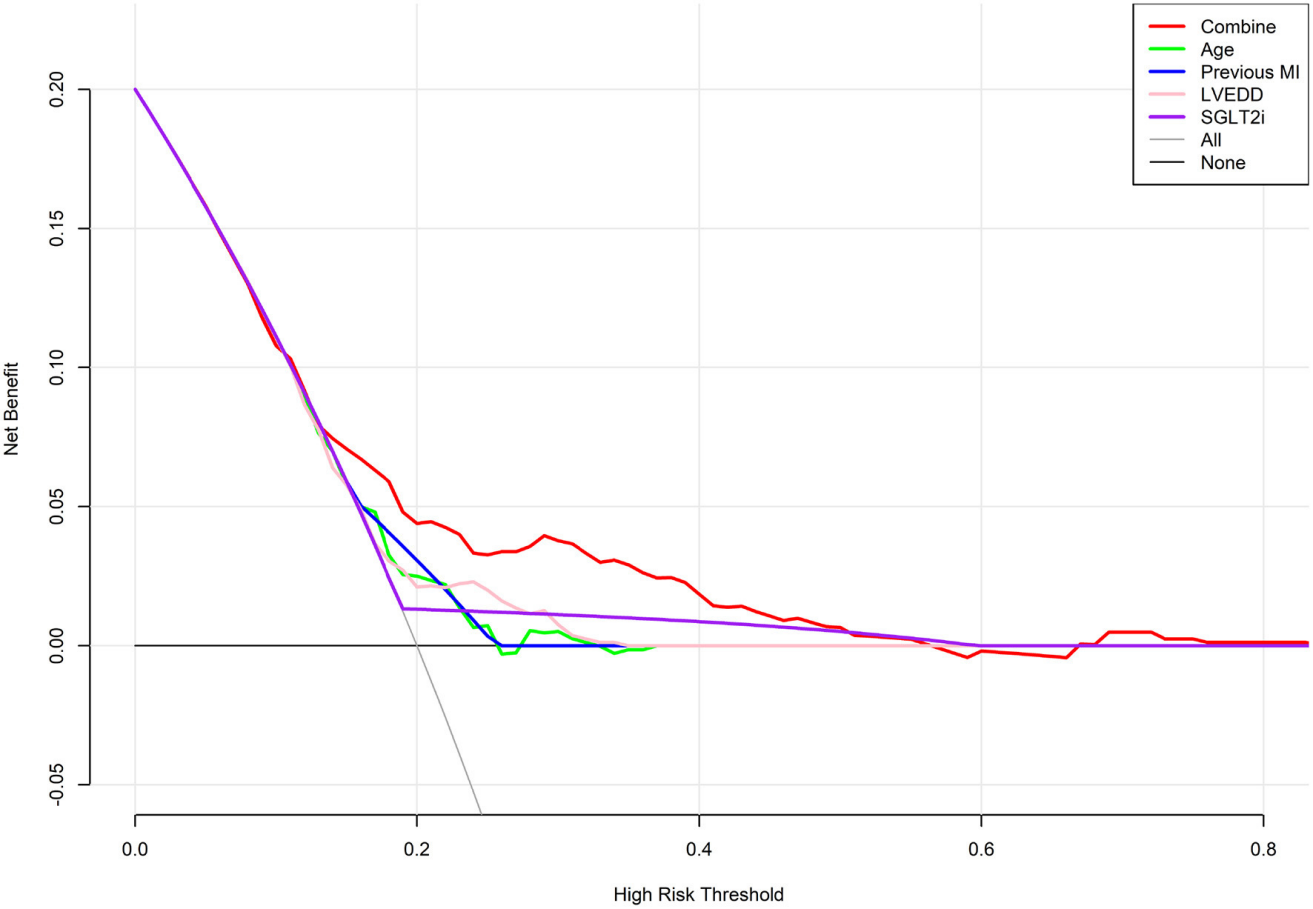
Decision curve analysis for predicting HFiEF. The final complex model outperformed single indices in terms of the net benefit rate.

DCA was performed to determine the net clinical benefit of the predictive model. It showed that compared to each factor, the nomogram predicted the occurrence of HFiEF 1 year after successful CTO PCI in HF patients more accurately (Figure 5).

## Discussion

In this retrospective study, we created and validated a simple model to predict HFiEF occurrence 1 year after successful CTO PCI in chronic HF patients without severe residual stenosis, as measured by an rSS of ≤ 8. The nomogram, comprising four variables (age, previous MI, LVEDD, and SGLT2i) enables clinical stratification of candidates for revascularization to achieve a better prognosis. The C-index value was 0.666 (95% CI, 0.613–0.719), indicating a moderate level of predictive ability, and the calibration curve exhibited good consistency.

This is the first study to construct a user-friendly model predicting HFiEF 1 year after successful CTO PCI in patients with HF. In our cohort, 35.5% of patients experienced a substantial improvement in LVEF 1 year after successful CTO PCI. To address multiple collinearity among variables and avoid overfitting, LASSO regression was used for initial variable selection. Younger age, absence of myocardial infarction history, smaller LVEDD, and SGLT2i use were more likely to achieve HFiEF 1 year after successful CTO PCI. Notably, the advantage of our nomogram is that all variables are readily available in the patient's medical history and echocardiographic results.

### Age and HFiEF

In CTO patients with HF, prior studies have revealed that successful CTO PCI contributes to a statistically significant improvement in LVEF (15). However, the relationship between age and HFiEF in subjects with CTO and HF is unclear. Consistent with numerous reports, the current study identified age as an independent predictor of HFiEF in patients with HF (16–18). Li et al. reported that age was a powerful independent predictor of HFiEF over 6 months of follow-up among 447 patients with HF (16). Similarly, Park et al. found that patients with HFiEF were younger than those without (18). Furthermore, animal experiments suggested that aging animals showed persistent tissue fibrosis and cardiac dysfunction with impaired reverse remodeling, unlike younger animals that experienced complete cardiac function recovery (19). Older HF patients carry a higher risk of comorbidities such as diabetes mellitus, hypertension, and anemia, and are prone to significant calcification and multiple and diffuse lesions (11, 20). In addition, functions and physiological processes degrade to different degrees with age. The aging myocardium is more susceptible to pathological damage due to decreased mitochondrial function and myocardial regeneration (21–23).

### Previous MI and HFiEF

MI is characterized by myocardial damage and apoptosis. MI triggers neuroendocrine regulations and an inflammatory response, resulting in changes in the morphological structure of the myocardium, ultimately affecting diastolic and systolic function (24, 25). Wilcox et al. determined that patients who suffered from MI were far less likely to experience HFiEF at 2 years (26). Choi et al. revealed that CTO PCI yielded favorable outcomes in non-MI HF patients (27). A good amount of dysfunctional, yet potentially salvageable myocardium may exist in these patients, thus being associated with the recovery of LV function after successful CTO revascularization and extensive stenotic lesion removal. However, for patients with MI, a large infarct size could result in adverse LV remodeling, leading to heart failure. Moreover, cardiac function recovery is unlikely to occur in patients with irreversible damage from MI (28). Importantly, the infarct size is closely related to the occurrence of adverse remodeling and functional impairment. Small infarcts are insufficient to lead to adverse remodeling (29).

### LVEDD and HFiEF

Clinicians also pay close attention to LV size parameters, such as LVEDD, which largely reflect LV remodeling in HF. Previous reports have demonstrated that patients with a larger LV size have a higher risk of subsequent deterioration in LVEF (16, 17, 30). Park et al. identified that a larger LVEDD at baseline led to the recurrence of HF in individuals with idiopathic dilated cardiomyopathy (17). In the current study, LVEDD was the most significant predictor of HFiEF. After LASSO analysis, we found that smaller LVEDD at baseline was an independent factor that predicted a greater likelihood of improved LVEF than the other echocardiographic parameters, including LVEF at baseline. Our study underlined the clinical significance of LVEDD. Adverse remodeling persisted and steadily worsened due to ongoing myocardial injury, resulting in LV chamber dilatation. To some extent, LVEDD reflects the degree of LV remodeling. A larger LVEDD may intensify the disruption of the myocardial ultrastructure and represents more severe adverse cardiac remodeling, leading to a potentially lower cardiac function recovery (28). Therefore, it is essential to provide early revascularization, optimize medical therapy, and reinforce postoperative follow-up to reverse or alleviate cardiac remodeling and achieve good prognoses for these patients.

### SGLT2i and HFiEF

An important finding in our study was that patients prescribed with SGLT2i had a greater odds of being in the HFiEF group than the other drugs we collected, such as angiotensin receptor neprilysin inhibition (ARNI), beta-blocker, and ivabradine. SGLT2i worked by inhibiting SGLT2i in the proximal convoluted tubule to prevent glucose reabsorption and facilitate its excretion in urine (31). Recent studies have found that SGLT2i showed cardioprotective actions independent of the hypoglycemic effects in patients with and without type 2 diabetes mellitus (T2DM) (32–34). The DAPA-HF trial revealed that dapagliflozin significantly reduced HF hospitalization and cardiovascular mortality, independent of the presence of T2DM (32). However, the exact mechanism was not fully understood, and SGLT2i might have contributed to reducing generalized congestion and intravascular volume, thus decreasing cardiac afterload and preload (35). In contrast, previous studies have revealed that beta-blocker was a predictor of HFiEF (16, 18). Notably, details regarding the medications were not inadequate in many prior studies. Furthermore, the evidence of new classes of drugs for HF has been growing over the last few years. Therefore, we considered more details regarding medications, and demonstrated that SGLT2i use was a positive predictor of HFiEF in CTO patients with HF.

Otherwise, after LASSO analysis, although eGFR was selected as a candidate for further construction of the prediction model, it showed no statistical significance after multivariate logistic regression analysis. Considering the results of previous reports and the clinical significance of eGFR (14), we believed it may have a potential effect on the improvement of LVEF. Our follow-up duration may have been relatively short to draw concrete conclusions, and we plan to conduct a prospective and longer follow-up trial in the future.

## Limitations

Our investigation has several limitations. First, due to the single-center retrospective nature of the study, selection bias was unavoidable, and the results may not be representative of the entire population of Chinese CTO patients with HF, though we have attempted to provide detailed information in our study. Second, our study lacked an external population to validate the predictive model. However, the internal validation was relatively good, which is a strength of our study. Finally, although we tried to collect detailed variables, some potentially critical prognostic factors, such as medication changes after discharge, could not be collected.

## Conclusions

In conclusion, we built a scoring model to predict the possibility of HFiEF 1 year after successful CTO PCI in patients with HF. The findings provide a practical and easy-to-use reference tool for clinicians to select suitable PCI candidates to improve cardiac function.

## Data Availability Statement

The raw data supporting the conclusions of this article will be made available by the authors, without undue reservation.

## Ethics Statement

The studies involving human participants were reviewed and approved by the Scientific Research and Clinical Trial Ethics Committee of The First Affiliated Hospital of Zhengzhou University. Written informed consent for participation was not required for this study in accordance with the national legislation and the institutional requirements.

## Author Contributions

LY, HL, and GG contributed to the statistical analysis, interpretation of the data, and prepared figures and/or tables. LY wrote the manuscript. JD, ZH, LK, and HS were helpful for data collection. YZ and XW supervised the project and revised the draft of the article. All authors contributed to the article and fully accountable for the content of the manuscript.

## Conflict of Interest

The authors declare that the research was conducted in the absence of any commercial or financial relationships that could be construed as a potential conflict of interest.

## Publisher's Note

All claims expressed in this article are solely those of the authors and do not necessarily represent those of their affiliated organizations, or those of the publisher, the editors and the reviewers. Any product that may be evaluated in this article, or claim that may be made by its manufacturer, is not guaranteed or endorsed by the publisher.
